# Hygrometry of gas delivered with different high flow oxygen humidifiers and impact on comfort of the level of hygrometry

**DOI:** 10.1186/2197-425X-3-S1-A805

**Published:** 2015-10-01

**Authors:** F Lellouche, PA Bouchard

**Affiliations:** Institut Universitaire de Cardiologie et de Pneumologie de Québec, Lac-Beauport, Canada; Institut Universitaire de Cardiologie et de Pneumologie de Québec, Québec, Canada

## Introduction

The high flow oxygen therapy is increasingly used in patients with acute respiratory failure. However, an adequate humidification is necessary to improve patient tolerance to this therapy. Few data is available in literature about the hygrometry delivered by these devices.

## Objectives

To evaluate hygrometric performances of several high flow oxygen therapy devices and to evaluate the potential clinical impact of different levels of hygrometry.

## Methods

We have tested on a bench the hygrometric performance of different oxygen high flow therapy systems (OptiFlow/Airvo - Fisher&Paykel, Hydrate OMNI - Hydrate inc., Precision flow - Vapoterm,). We have used psychrometric method to measure hygrometry.

For each condition, we made 3 hygrometric measurements at different moments of the day. The measurements were taken proximal to the simulated patient's interface. We have tested different settings (intubation and NIV settings for the Optiflow), different flow rates (10, 20-30-40 and 60l/min) and two ambient temperatures: normal temperature (22-24°C) and high temperature (28-30°C). We evaluated the comfort in 10 healthy subjects blinded for different humidity levels (20, 30 and 40 mgH_2_O/L) during 10 minutes in random orders.

## Results

At normal temperature (22-24°C), the Fisher&Paykel Optiflow system (with or without compensation algorithm) provided the highest absolute humidity (39.3 vs. 35.9 vs. 32.0 for F&P, Hydrate and Vapotherm, p = 0.0009), regardless of the used flow rate (hygrometric performance > 37 mgH_2_O/L). However, with the NIV setting, humidity delivered was low (between 20 and 25 mgH2O/L). At high temperature (28-30°C), the Precision flow -Vapotherm system provided the highest absolute humidity at lower flow rates (10 and 20 l/min) (31.8 vs. 32.2 vs. 37.2, for F&P, Hydrate and Vapotherm, p = 0.001). However, at high flows, performances were significantly reduced with the Vapotherm device (37.2 vs.31.8 for 10-20L/min and 30-40L/min, p = 0.027).

In healthy subjects, comfort was equivalent at 30 and 40 mgH2O/L but significantly lower at 20 mgH2O/L.

The main results are presented on the following figures.

## Conclusions

With NIV setting, Optiflow delivered low humidity, with lower comfort in the short term evaluation in healthy subjects. In the other conditions, the majority of the tested humidification systems deliver at least 30mgH_2_O/L. At high temperature (28-30°C), the majority of the systems showed a decrease in their hygrometric performance (except Precision flow). The flow rate influence is moderated regarding the humidification performance (except for Vapotherm). The new technology Hydrate performed reasonably well within the tested conditions.

There is no clear recommendations for the level of humidity to deliver during HFOT, but NIV setting may be less comfortable.

**Figure 1:**
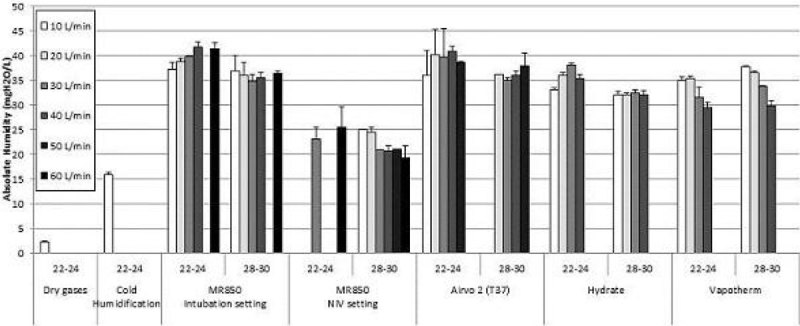
**Hygrometric performances**.

**Figure 2:**
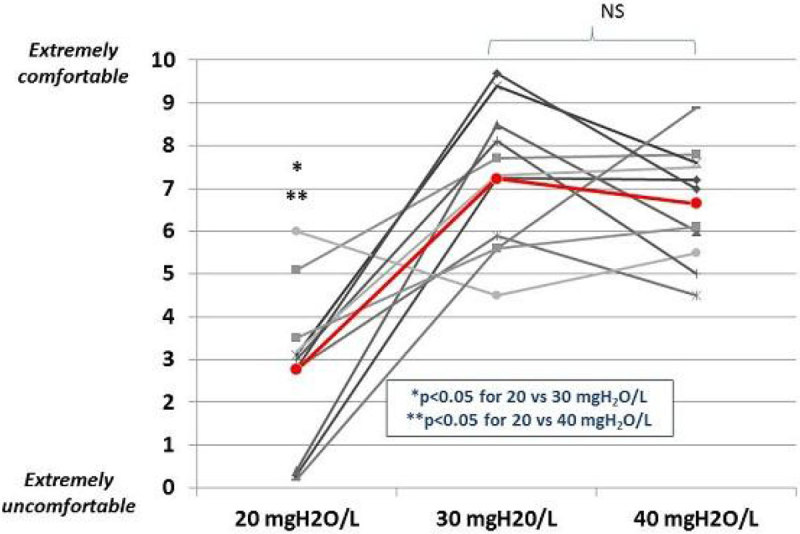
**Comfort at 20-30-40 mgH2O/L**.

